# Supplementary material for: Quantum chemical molecular dynamics and metadynamics simulation of aluminium binding to amyloid-β and related peptides
Source: R Soc Open Sci. 2020 Feb 5;7(2):191562. doi: 10.1098/rsos.191562 (PMC7062105; doi:10.1098/rsos.191562)
Supplement: Combined document uploaded [file rsos191562supp1.docx]

**Supporting Information for “Quantum Chemical Molecular Dynamics and Metadynamics Simulation of Aluminium Binding to Amyloid-β and Related Peptides”**

|  |  |
| --- | --- |
|  |  |

**Figure S1** Temperature and total energy of **Al-AADAA** over the course of 1 ns MD simulation. Top: 2 fs timestep with bonds to hydrogen restrained; Bottom: 4 fs timestep with all bonds except Al-O restrained.

|  |
| --- |
|  |

**Figure S2** Potential energy and RMSD of **Al-AADAA** initial structure from 1 ns MD simulation with 4 fs timestep

|  |
| --- |
|  |

**Figure S3** Temperature and total energy of **Al-AADAA** over the course of 100 ps metadynamics simulation.

|  |
| --- |
|  |
|  |

**Figure S4** RMSD against time for Al-EAAAD from a) conventional MD, b) metadynamics with
ki/N = 0.01, c) with ki/N = 0.025

**Figure S5** Backbone RMSD against time for Al-AB16 conventional MD

| 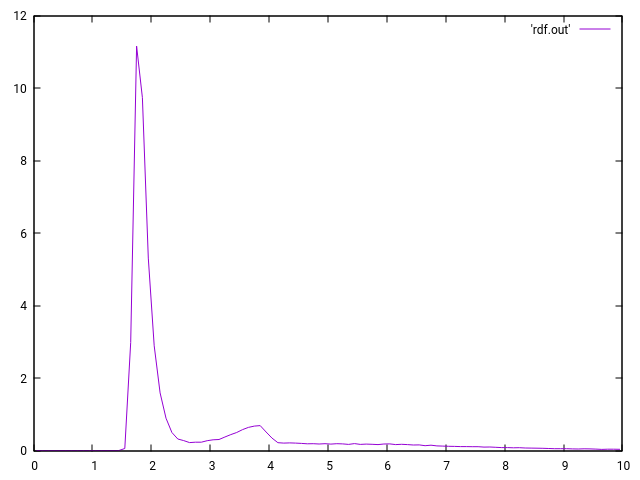 | 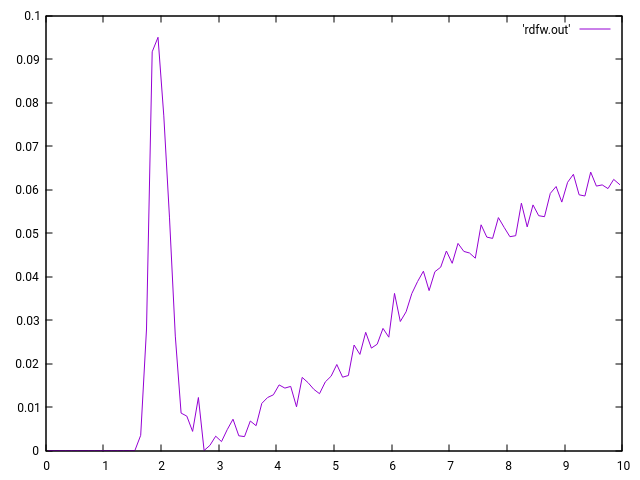 |
| --- | --- |
| 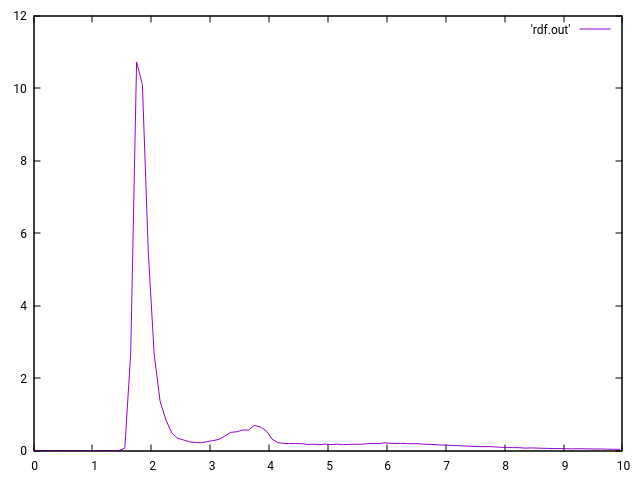 | 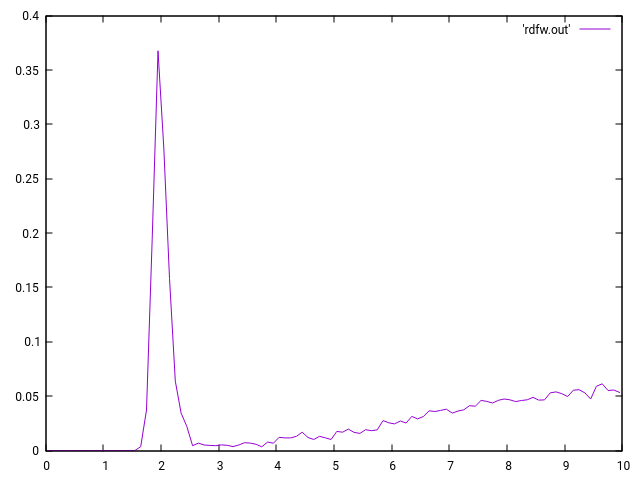 |
| 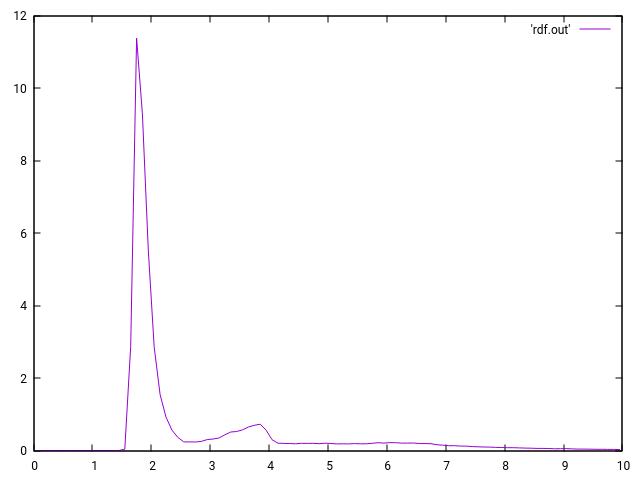 | 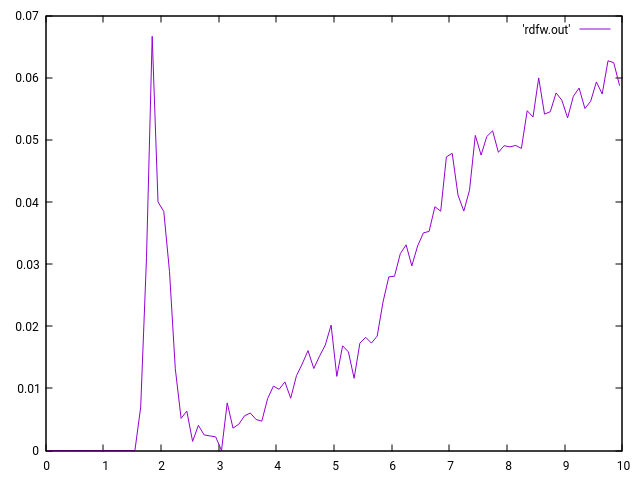 |

**Figure S6** Radial distribution functions Al-peptide (left) and Al-water (right) contacts from three independent metadynamics simulation of **Al-AB16**. The combined g(r) is shown in main text.

**Figure S7** Coordination number of Al vs simulation time for two independent metadynamics simulation of **Al-AB16**.
